# Presence of heterocyclic amine carcinogens in home-cooked and fast-food camel meat burgers commonly consumed in Saudi Arabia

**DOI:** 10.1038/s41598-017-01968-x

**Published:** 2017-05-10

**Authors:** Mohammad Rizwan Khan, Mu Naushad, Zeid Abdullah Alothman

**Affiliations:** Department of Chemistry, College of Science, King Saud University, P.O. Box 2455, Riyadh, 11451 Saudi Arabia

## Abstract

Heterocyclic amines (HCAs) are formed by cooking protein-rich foods, for instance, meat and fish, and are listed as *possible human carcinogens*. In the present study, the presence of five potential HCAs (IQ, MeIQ, MeIQx, 4,8-DiMeIQx, and PhIP) in cooked camel meat burgers was analyzed for the first time. The analysis was performed in home-cooked and fast-food burger samples containing food additives. The applied cooking technique for the home-cooked samples was pan frying for a controlled cooking time and temperature. In the control cooked meat samples (samples that contained no food additives), the concentrations of MeIQx, 4,8-DiMeIQx, and PhIP ranged from 2.47 ng/g to 4.89 ng/g, whereas IQ and MeIQ were found to be below the limit of quantification. The concentrations contents of MeIQx, 4,8-DiMeIQx, and PhIP in the home-cooked and fast-food samples ranged from 1.52 ng/g to 2.13 ng/g and 1.85 ng/g to 3.46 ng/g, respectively. IQ and MeIQ were not detected in either type of sample. In comparison to the control samples, the home-cooked and fast-food samples produced lower levels of HCAs. Such observations could result from the existence of antioxidants in incorporated food additives, which induce pro-oxidative effects with the successive formation and/or scavenging of free radicals.

## Introduction

Humans are incessantly exposed to unpredictable levels of hazardous chemicals in food, water, and air^1^. Human exposure to such chemicals can contribute to the development of various cancers^2^. Heterocyclic amines (HCAs) are known to be foodborne mutagens/carcinogens, detected at low concentrations (parts per billion) in food containing protein-rich meat or fish processed under household cooking conditions^1, 3–5^. The levels of HCAs produced in meat products are mainly dependent on the kinds of meat product, cooking time, and temperature^6, 7^. To date, more than twenty-five HCAs have been identified in cooked meat products, and they are usually formed from a nonenzymatic chemical reaction (Maillard reaction) between reducing sugars and amino acids that occurs under normal cooking conditions^8–10^. The intake of HCAs through our diet (especially the consumption of cooked meat) has also been considered a cause of various cancers^11–15^. A number of epidemiological studies have shown a relationship between high cooked-meat intake and the risk of developing cancer^11–15^. Previously published studies have reported positive relationships between a high cooked-meat intake and the development of several cancers, such as bladder^16^, colorectal^17, 18^, breast^19^, colon, rectum, kidney^20^, and prostate cancer^21^.

Based on such findings, the International Agency for Research on Cancer (IARC) has categorized 2-amino-1-methyl-6-phenylimidazo[4,5-b]pyridine (PhIP), 2-amino-3,8-dimethylimidazo[4,5-f]quinoxaline (MeIQx), and 2-amino-3,4-dimethylimidazo[4,5-f]quinoline (MeIQ) as *possible human carcinogens*, whereas 2-amino-3-methylimidazo[4,5-f]quinoline (IQ) has been categorized as a *probable human carcinogen* ^22^. Recently, the National Toxicology Program (NTP) also listed PhIP, MeIQx, MeIQ, and IQ as *reasonably anticipated to be a human carcinogen* ^23^. To illustrate the characteristics of real HCA exposure in humans, numerous investigations have examined meat products either thermally processed at home or from fast-food outlets. HCA concentrations in home-cooked meat products have been quantified in numerous studies in Saudi Arabia^5^, Brazil^24^, USA^25^, Singapore^26^, Spain^3, 27^, Denmark^28^, Switzerland^29^, and Korea^30^. HCA concentrations in fast-food meat products have also been quantified in a number of investigations in Sweden^31^, USA^32–34^, Thailand^34^, Canada^35^, and the United Kingdom^36^. These studies have produced valuable data relating to the HCA levels in meat products and can act as important markers from a community health perspective. In addition, these data can also offer information on aspects that affect HCA occurrence and specify means of diminishing or removing such carcinogens.

Camel meat is one of the most commonly eaten meats in the Saudi Arabian diet and, thus, could be a significant source of HCA exposure. Over recent years, fast food has become one of the main channels in the food consumer service industry, especially in Saudi Arabia, where fast-food sales in 2015 totaled 22.6 billion Saudi riyal^37^. Among the types of fast food, burger meat consumption has grown by 11% due to due to its remarkable appeal to the public^37^. Therefore, the study of the relationship between HCAs and their role in the etiology of human cancer requires the precise determination of HCAs in such meat products. The objectives of the present investigation were to determine, for the first time, the formation of HCAs in camel meat burgers, to reveal new potential sources of HCAs, and to determine how the concentrations and kinds of HCA could be affected by the addition of food additives typically used in cooking methods in Saudi Arabia.

## Results and Discussion

**Figure 1:**
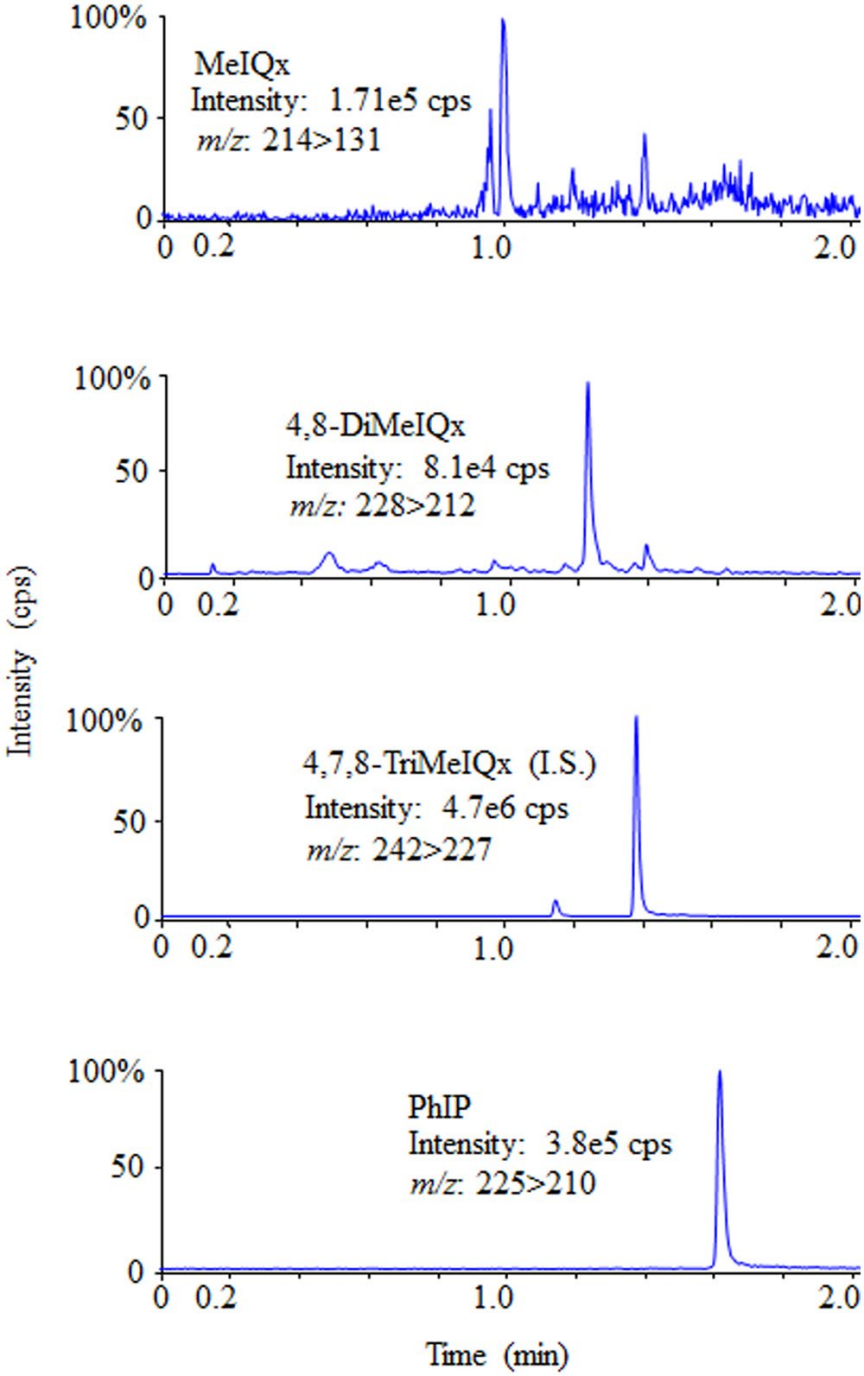
UPLC-MS/MS chromatograms of HCAs in home-cooked burger.

In this study, two types of camel meat burger (home- cooked and fast- food) were analyzed for five potential HCAs. Only the superficial layer of each meat sample was studied since HCAs primarily occur in this layer. The quantity of HCAs obtained in the superficial meat layer was converted into the quantity of HCAs in each entire sample by considering the ratio between the weight of the superficial meat layer and that of the whole meat sample. To check the performance of the method, quality control parameters, such as the linearity, limit of detection (LOD, signal-to-noise ratio 3:1), limit of quantification (LOQ, signal-to-noise ratio 10:1), and recovery values, were studied. The obtained LOD and recovery values are detailed in Table 1. The LOD values ranged between 0.01 ng/g and 0.03 ng/g, while the recovery values ranged from 54% to 65%. The LOD and recovery values were found to be in good agreement with those previously obtained from cooked beef and camel meat products^5, 27, 38, 39^. The LOD and recovery values in beef and camel meats were comparable, which may be due to the similarity of the meat composition^5^ and of the SPE techniques applied, with the exception of the extraction solvent being dichloromethane instead of ethyl acetate^39, 40^. Calibration curves for the studied HCAs were constructed and found to be linear, with correlation coefficients (*r* ^*2*^) greater than 0.998. Table 2 illustrates the mean values of the HCAs and their equivalent standard deviations achieved from three standard addition calibrations spiked at different levels. In the control samples, the signal-to-noise ratio values for IQ and MeIQ were found to be <10; thus, the outcomes were demonstrated to be below the limit of quantification. Furthermore, IQ and MeIQ were not detected in either the home-cooked or fast-food burger samples, and the results indicate the two as not detected. As an example, Fig. 1 displays the UPLC-MS/MS chromatograms of the HCAs in the home-cooked burger samples. It can be seen that the detected concentrations of HCAs varied substantially: whereas PhIP was detected at high concentrations, MeIQx and 4,8-DiMeIQx were detected at lower concentrations. Chromatograms relating to IQ and MeIQ are not presented because they were either not detected or found to be below the limit of quantification in the analyzed samples. It can also be seen that good UPLC-MS/MS sensitivity was attained during HCA determination, while six SRM transitions from the triple quadrupole instrument were acquired at identical times.

**Table 1:** Recovery rates (R) and limit of detection (LOD) of HCAs in cooked camel meat burgers.

Camel meat	IQ		MeIQ		MeIQx		4,8-DiMeIQx		PhIP	
	R (%)	LOD (ng/g)	R (%)	LOD (ng/g)	R (%)	LOD (ng/g)	R (%)	LOD (ng/g)	R (%)	LOD (ng/g)
Control^a^	63	0.01	65	0.01	61	0.01	63	0.01	57	0.02
Home-cooked	58	0.02	60	0.03	56	0.02	56	0.03	54	0.03
Fast-food outlets	57	0.02	61	0.03	57	0.02	58	0.02	55	0.02

^a^Meat cooked without food additives.

**Table 2:** Levels of HCAs^a^ (ng/g ± SD) in cooked camel meat burgers.

Camel meat	IQ	MeIQ	MeIQx	4,8-DiMeIQx	PhIP	Total HCAs
Control^b^	nq	nq	2.47 ± 0.10	2.28 ± 0.11	4.89 ± 0.82	9.64
Home-cooked	nd	nd	1.52 ± 0.14	1.33 ± 0.15	2.13 ± 0.11	4.98
Fast-food outlets^c^	nd	nd	1.85 ± 0.12	1.66 ± 0.12	3.46 ± 0.93	6.97

^a^Mean of HCAs; SD: standard deviation attained from standard addition calibration curve; ^b^meat cooked without food additives; nq: below quantification limit; nd: not detected; ^c^sequence analysis data of fast-food outlets samples have been provided in the supplementary information.

Typically, MeIQx, 4,8-DiMeIQx, and PhIP were detected most often in the studied samples, whereas IQ and MeIQ were only detected in the control samples. Among them, PhIP was produced at high concentrations, ranging from 2.13 ng/g to 4.89 ng/g. However, MeIQx and, 4,8-DiMeIQx were produced at lower concentrations, ranging from 1.33 ng/g to 2.47 ng/g. The values obtained for MeIQx, 4,8-DiMeIQx, and PhIP in the control meat samples were found to be in good agreement with those achieved in previous studies^5, 38^. Generally, IQ and MeIQ were either not detected or generated at very low concentrations in the meat samples, which might be because elevated thermal processing temperatures are necessary for their formation^41^. In contrast, MeIQx and 4,8-DiMeIQx are typically formed at higher concentrations under household cooking conditions^41^. PhIP is usually found at higher concentrations in such types of meat, and the concentrations found in the current study were in good agreement with those reported by previous studies^34, 42^. Nonetheless, in other high-protein foods, such as cooked chicken and fish (swordfish), PhIP was found in very high concentrations (>100 ng/g), especially in swordfish^43, 44^. The HCA levels in such foods are higher than those detected in the present study and these food products are typically defined as being extremely HCA contaminated^43, 44^. In addition, PhIP could also be the main HCA in the human diet.

Compared with the control samples, lower concentrations of HCAs were formed in the home-cooked and fast-food samples. MeIQx, 4,8-DiMeIQx, and PhIP were detected in both the sample types at concentrations ranging from 1.33 ng/g to 3.46 ng/g cooked meat, while IQ and MeIQ were not produced in any of the burger samples. These results illustrate that the addition of food additives in both the home-cooked and fast-food samples was sufficient to reduce HCA formation. It can also be observed from Table 2 that the home-cooked samples produced relatively lower concentrations of HCAs than the fast-food samples, which may be due to either the longer cooking time and higher temperature or the food additives incorporated during food preparation^45, 46^. In previous studies, authors reported lower concentrations of HCAs in meat burgers than in other thermally processed meat products, signifying that the use of food additives diminished the formation of HCAs^27^. Khan *et al*. described the effect of food additives on the formation of HCAs in cooked meat products and found a large reduction in HCAs^38^. For instance, the addition of garlic, ginger, pepper, tomato, and onion reduced the amounts of MeIQx (50–76%), 4,8-DiMeIQx (47–80%), and PhIP (45–78%)^38^. A number of researchers have determined that plants of the genus *Allium* have positive antioxidant effects^45, 47, 48^, likely because of their numerous antioxidant constituents, such as polyphenolics and flavonoids^45^. This study was the first to determine the HCA concentration in cooked camel meat burgers, which are frequently eaten in Saudi Arabia. Overall, the results revealed that the concentration of HCAs changed with the addition of food additives, as well as with the cooking time and temperature.

## Conclusions

The concentrations of five potential HCAs were identified for the first time in camel meat burgers, either home- cooked or from fast-food outlets in Saudi Arabia. The results demonstrated that HCAs were detected in all of the analyzed burger samples. The concentrations of HCAs in the home-cooked and fast-food burgers were lower than in the control burger samples. In the latter, the concentrations of MeIQx, 4,8-DiMeIQx, and PhIP ranged from 2.47 ng/g to 4.89 ng/g, whereas the concentrations of IQ and MeIQ were found to be below the limit of quantification. By contrast, the concentrations of MeIQx, 4,8-DiMeIQx, and PhIP in the home-cooked and fast-food burger samples were relatively lower, ranging from 1.52 ng/g to 3.46 ng/g. IQ and MeIQ were not detected in either type of sample. Such variations could be due to the applied cooking time and temperature, as well as the existence of antioxidants in incorporated food additives, which play an important role in the formation of HCAs. The data obtained from the cooked camel meat burgers could be applied to measure HCA exposure, especially in the Saudi Arabian population, and could be used in epidemiology studies.

## Materials and Methods

### Chemicals and reagents

HPLC-grade ethyl acetate, methanol, and acetonitrile were obtained from Merck (Darmstadt, Germany). Formic acid (98%), ammonium formate, and ammonium acetate were purchased from Merck (Darmstad, Germany). Sodium hydroxide and ammonia solution (25%) were purchased from BDH Laboratory Supplies (Poole, UK) and Panreac Química (Barcelona, Spain), respectively. All chemicals were of analytical/reagent grade.

**Figure 2:**
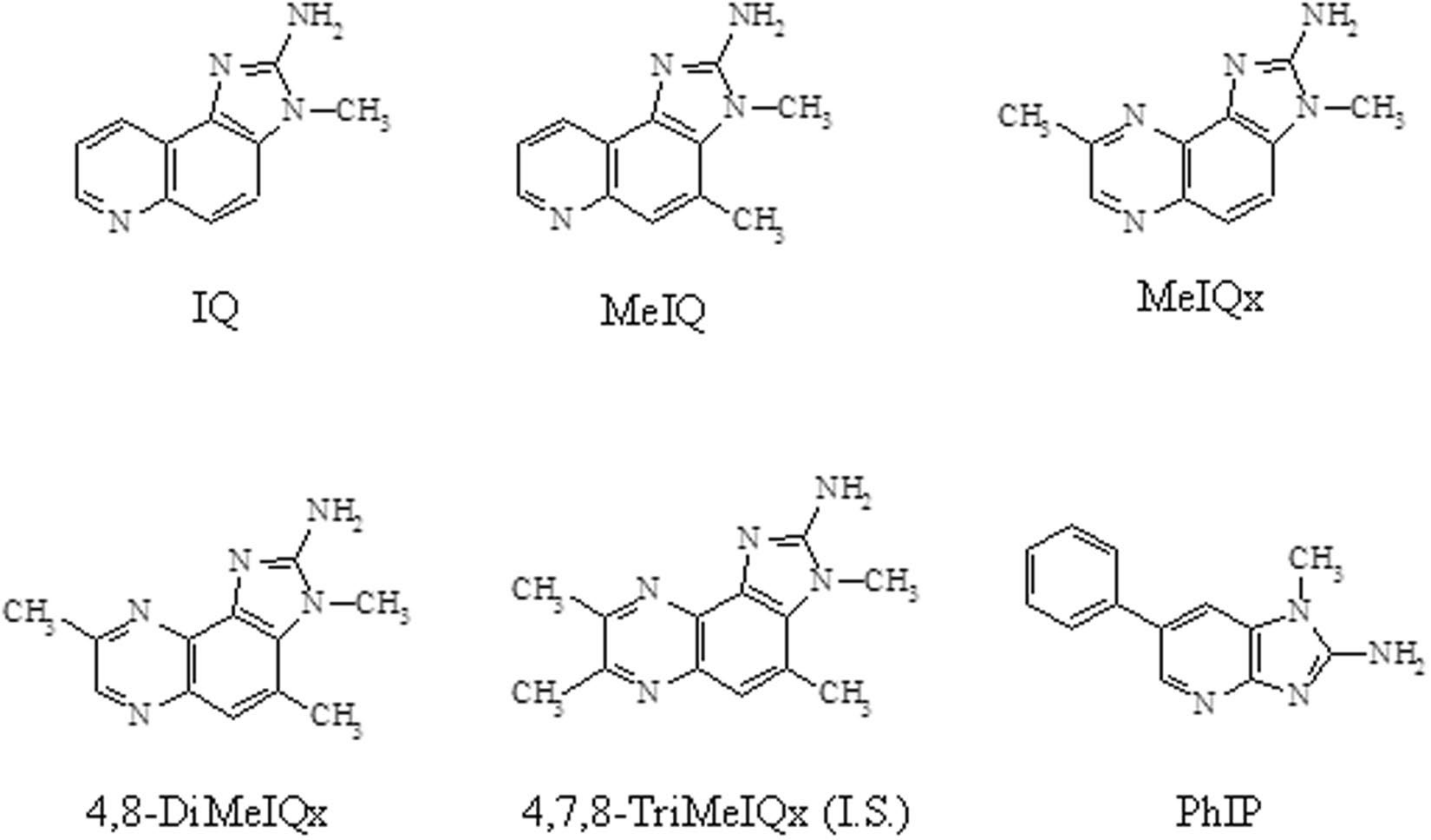
Structures and abbreviations of studied heterocyclic amines.

The HCAs 2-amino-3-methylimidazo[4,5-f]quinoline (IQ), 2-amino-3,4-dimethylimidazo[4,5-f]quinoline (MeIQ), 2-amino-3,8-dimethylimidazo[4,5-f]quinoxaline (MeIQx), 2-amino-3,4,8-trimethylimidazo[4,5-f]quinoxaline (4,8-DiMeIQx), 2-amino-1-methyl-6-phenylimidazo[4,5-b]pyridine (PhIP), and 2-amino-3,4,7,8-tetramethyl-imidazo [4,5-f]quinoxaline (4,7,8-TriMeIQx) (Fig. 2) were obtained from Toronto Research Chemicals (Toronto, Canada). 4,7,8-TriMeIQx was used as an internal standard (I.S.), and the purity of the HCAs was >99%.

Water purification was performed using a Milli-Q water purification system, model Advantage A10 (Millipore Corporation, Bedford, USA). Stock standard solutions of each HCA at a level of 150 µg/g were prepared in organic solvent (methanol) and used for additional dilutions. Standard mixed solutions of the HCAs at levels ranging from 0.003 µg/g to 1.0 µg/g, containing the internal standard 4,7,8-TriMeIQx (0.5 µg/g), were prepared by weight to establish a linear range over which to construct the calibration curves. Before being injected into the ultra-performance liquid chromatography (UPLC) system, the HCA solutions and samples were filtered through a 0.45-µm PTFE filter (Macherey-Nagel GmbH, Düren, Germany). Octadecylsilane C_18_ (100 mg), Bond Elut propylsulfonyl silica PRS (500 mg) solid-phase extraction (SPE) cartridges, stopcocks, and coupling pieces were obtained from Varian (Harbor City, USA). An Extrelut NT20 extraction column was purchased from Merck (Darmstadt, Germany). Inert diatomaceous earth material was supplied from Agilent Technologies (Apple Valley, USA). Glass vials (40 mL) with screw caps containing a PTFE seal (Thermo Scientific, Rockwood, USA) were used to hold the meat samples.

To assess the cooking temperature of the camel meat, an insulated type K wire probe with the Normadics TC6 software (Cole-Parmer, Vernon Hills, USA) was used. Ultra-Turrax® T25 digital (IKA^®^, Staufen, Germany) and Microtron® MB800 (Kinematica AG, Littau, Switzerland) mixers were used to blend the heat-treated meat samples. For solid-phase extraction (SPE) and solvent evaporation, Visiprep™ and Visidry™ vacuum manifolds (Supelco, Gland, Switzerland) were applied.

### Sample cooking conditions

Twenty camel meat burger samples were obtained from various local fast-food outlets in Riyadh, Saudi Arabia. The samples were ground with a food processor (Microtron MB800), sieved, bottled, and stored at −30 °C until analysis. For the preparation of the three control and three home-cooked burger samples, fresh camel meat and food additives were purchased from a local store in Riyadh, Saudi Arabia. The meat was ground and prepared in 50-g patties that were 5 cm in diameter and 0.5 cm in thickness. However, for the fast-food samples, the size varied. A detailed description of the camel meat burger preparation is provided in Table 3. In brief, the meat patties were pan fried on a non-stick frying pan (Tefal, Paris, France) using a Nippotec electric stove (Shanghai, China). To measure the cooking temperature of the meat patties, five insulated type K wire probes with the Normadics TC6 software were used. The insulated wire probes were applied to the center of the pan, to the lower and upper sides of the patty, between the pan and the patty, and in the center of the patty. The frying temperature of the meat samples ranged from 215 °C to 225 °C, the total cooking time of the patties was 4 min (2 min on each side). The temperature of the patties was permitted to decrease to ambient temperature after cooking. Then, the weight loss of the thermally processed patties was measured as the variance between the quantity of the meat sample before and after the cooking process (Table 3). The superficial layer of the cooked meat, approximately 2–3 mm, was separated, ground, sieved, bottled, and stored at −30 °C until HCA extraction.

**Table 3:** Description of camel meat burger preparation.

Meat sample	Recipes	Cooking system	Cooking time (min/side)	Cooked meat (g)	Weight loss (%)
Control*	Ground camel meat (500.89 g) and, bread crumbs (50 g) combined together and blend gently using a stand mixer model MK-GB1WTZ (Panasonic, Shanghai, China). Then, the foods were prepared in the form of small patties followed by pan frying (4 min) and removed.	Pan frying	4	295.32	41.04
Home-cooked	Ground camel meat (500.34 g), cumin powder (1 g), bread crumbs (50 g), ginger powder (1 g), black pepper powder (0.5 g), cinnamon powder (0.5 g), coriander powder (0.5 g), red pepper powder (0.5 g), cloves powder (0.5 g) and table salt (1 g). Meat including food additives combined together and blends gently using a stand mixer model MK-GB1WTZ (Panasonic, Shanghai, China). Then, the foods were prepared in the form of small patties followed by pan frying (4 min) and removed.	Pan frying	4	288.65	42.30
Fast-food outlets	Camel meat.	Pan frying	—	—	—

*Meat products cooked without food additives; —not described.

### HCA extraction

The extraction and purification of HCAs from the thermally processed camel meat samples were performed following formerly established SPE procedures^40, 49^. The refrigerated samples were removed and allowed to reach ambient temperature. Then, 3-g subsamples from the superficial layer meat samples were homogenized with a solution of sodium hydroxide (1 M) using an Ultra-Turrax® T25 digital blender followed by mixing with inert diatomaceous earth material (13 g). The sample was moved to an Extrelut column coupled to a Bond Elut PRS (500 mg) cartridge, which was preconditioned successively with HCl (15 mL, 0.1 M), water (10 mL), and methanol (5 mL). An organic solvent (ethyl acetate, 75 mL) was used to extract the HCAs from the diatomaceous earth using a coupled Bond Elut PRS (500 mg) cartridge. After complete elution of ethyl acetate from the cartridge, the PRS cartridge was desiccated under vacuum using Visidry™ vacuum manifolds and washed successively with methanol/water (6:4, v/v, 15 mL) and water (2 mL). Subsequently, a Bond Elut C_18_ (100 mg) cartridge was preconditioned successively with methanol (15 mL) and water (5 mL), and coupled with the PRS cartridge. The desorption of HCAs from the PRS cartridge to the C_18_ cartridge was performed using an ammonium acetate (0.5 M, 20 mL, pH 8.5) solution. Lastly, the C_18_ cartridge was washed with water (5 mL) followed by vacuum desiccation. The HCAs were eluted from the C_18_ cartridge to a 1.5-mL Eppendorf tube (Wesseling-Berzdorf, Germany) with a methanol/ammonia (9:1, v/v, 0.8 mL) mixed solution. Using a nitrogen stream, the obtained solvent mixture containing the HCAs was evaporated until complete dryness. Once dried completely, the extract was dissolved with a methanolic solution (0.1 mL) containing the internal standard 4,7,8**-**TriMeIQx (0.5 µg/g). Lastly, the methanolic solution was passed through a PTFE syringe filter (0.45 µm) and stored at 4 °C before being injected into the UPLC-MS/MS system.

To overcome sample matrix effects, HCA quantification and recovery were carried out in triplicate using the standard addition method. The standard addition method included three spiked meat samples at different concentrations (50%, 200%, and 400%) and two non-spiked meat samples. The recovery values were measured from the linear regression slope attained from a graph of the spiked HCA amount versus the measured HCA amount. To obtain statistical data, analysis of variance (ANOVA) was used.

### HCA determination

Chromatographic separation of the HCAs was achieved on a rapid Acquity® UPLC technique equipped with a quaternary pump (Waters, Milford, USA). A reversed-phase Acquity® BEH C_18_ analytical column with dimensions of 50 mm × 2.1 mm i.d. and a 1.7 mm particle size (Waters, Milford, USA) was applied. A mixed solution of formic acid/ammonium formate (30 mM, pH 4.75) (A) and organic solvent acetonitrile (B) was used as a binary mobile phase at a flow rate of 1000 µL/min. The elution program of the mobile phase was as follows: 0–0.1 min, 5% B; 0.1–1.5 min, 5–30% B; 1.5–1.8 min, 30–60% B; 1.8–2.4 min, 60% B; 2.4–2.5 min, returned to its initial conditions; 2.5–3 min. Then, 10 µL of the sample was injected into the UPLC system^39^. To eliminate contamination during analysis, the column was rinsed with a mixture of methanol/water (50/50, v/v) for 10 min every 25 sample injections.

HCA detection was performed on a triple quadrupole mass spectrometer (MS/MS) (Micromass Quattro Premier, Milford, USA) fitted with an electrospray ionization (ESI) source. The mass spectrometer was operated in positive ionization mode. To acquire the UPLC-MS/MS data, selected reaction monitoring (SRM) was used. The optimized ESI source parameters were as follows: source temperature, 100 °C; desolvation temperature, 400 °C; cone voltage, 40 V; capillary voltage, 3.0 kV; cone gas, 49 L/h; desolvation gas, 804 L/h. A nitrogen generator model NM30LA (Peak Scientific, Inchinnan, UK) was used to supply the cone gas to the MS system. An argon gas cylinder was used to supply the collision gases to the MS system. Primary vacuum to the MS system was supplied using an Oerlikon rotary pump, model SOGEVACSV40BI (Cedex, France). The SRM parameters applied to the MS/MS system are given in Table 4. The MassLynx V4.1 software (Waters, Milford, USA) was used to obtain the UPLC-MS/MS data^39^.

**Table 4:** SRM parameters applied with MS/MS technique*.

HCAs	Precursor ion [M + H]^+^ (*m*/*z*)	Quantification		Confirmation**	
		Product ion (*m*/*z*)	Collision energy (eV)	Product ion (*m*/*z*)	Collision energy (eV)
IQ	199	184	30	157	35
MeIQ	213	198	25	197	30
MeIQx	214	199	30	131	25
4,8-DiMeIQx	228	213	30	187	25
4,7,8-TriMeIQx (IS)	242	227	25	201	30
PhIP	225	210	25	183	30

*Dwell time (25 ms) in all HCAs; **confirmation ion intensity was >10%; IS = Internal Standard.

## Electronic supplementary material

Supplementary Information
