# Supplementary material for: Presence of heterocyclic amine carcinogens in home-cooked and fast-food camel meat burgers commonly consumed in Saudi Arabia
Source: Sci Rep. 2017 May 10;7:1707. doi: 10.1038/s41598-017-01968-x (PMC5431862; doi:10.1038/s41598-017-01968-x)
Supplement: Supplementary file 1 — Supplementary Information [file 41598_2017_1968_MOESM1_ESM.pdf]

Presence of heterocyclic amine carcinogens in home-cooked and fast-food camel meat burgers commonly consumed in Saudi Arabia

Mohammad Rizwan Khan*, Mu Naushad, Zeid Abdullah Alothman

Table 1.

Levels of HCAs (ng/g ± SD) in camel meat burgers obtained from fast-food outlets

Burgers	IQ	MeIQ	MeIQx	4,8-DiMeIQx	PhIP	Total HCAs
Sample 1	nd	nd	1.78 ± 0.13	1.69 ± 0.12	3.41 ± 0.95	6.88
Sample 2	nd	nd	1.89 ± 0.11	1.71 ± 0.11	3.49 ± 0.92	7.09
Sample 3	nd	nd	1.85 ± 0.12	1.65 ± 0.12	3.50 ± 0.92	7.00
Sample 4	nd	nd	1.82 ± 0.12	1.66 ± 0.12	3.46 ± 0.93	6.94
Sample 5	nd	nd	1.93 ± 0.11	1.68 ± 0.12	3.44 ± 0.93	7.05
Sample 6	nd	nd	1.85 ± 0.12	1.58 ± 0.13	3.48 ± 0.92	6.91
Sample 7	nd	nd	1.82 ± 0.12	1.64 ± 0.12	3.53 ± 0.91	6.99
Sample 8	nd	nd	1.76 ± 0.13	1.62 ± 0.13	3.44 ± 0.93	6.82
Sample 9	nd	nd	1.88 ± 0.12	1.72 ± 0.11	3.42 ± 0.95	7.02
Sample 10	nd	nd	1.79 ± 0.13	1.67 ± 0.12	3.46 ± 0.93	6.92
Sample 11	nd	nd	1.85 ± 0.12	1.65 ± 0.12	3.48 ± 0.92	6.98
Sample 12	nd	nd	1.78 ± 0.13	1.69 ± 0.11	3.46 ± 0.92	6.93
Sample 13	nd	nd	1.86 ± 0.12	1.69 ± 0.11	3.43 ± 0.94	6.98
Sample 14	nd	nd	1.92 ± 0.11	1.63 ± 0.13	3.42 ± 0.95	6.97
Sample 15	nd	nd	1.75 ± 0.13	1.67 ± 0.12	3.41 ± 0.95	6.83
Sample 16	nd	nd	1.95 ± 0.11	1.65 ± 0.12	3.45 ± 0.93	7.05
Sample 17	nd	nd	1.86 ± 0.12	1.59 ± 0.13	3.52 ± 0.90	6.97
Sample 18	nd	nd	1.85 ± 0.12	1.67 ± 0.12	3.48 ± 0.92	7.00
Sample 19	nd	nd	1.94 ± 0.11	1.66 ± 0.12	3.42 ± 0.95	7.02
Sample 20	nd	nd	1.87 ± 0.12	1.68 ± 0.12	3.50 ± 0.92	7.05

SD: standard deviation attained from standard addition calibration curve; nd: not detected
